# Spectrophotometric Determination of Risedronate in Pharmaceutical Formulations via Complex Formation with Cu (II) Ions: Application to Content Uniformity Testing

**Published:** 2008-12

**Authors:** M. I. Walash, M. E.-S. Metwally, M. Eid, R. N. El-Shaheny

**Affiliations:** *Department of Analytical Chemistry, Faculty of Pharmacy, University of Mansoura, Mansoura, Egypt*

**Keywords:** spectrophotometry, risedronate, Copper (II) sulfate, complex formation, content uniformity

## Abstract

A simple, sensitive, rapid and accurate spectrophotometric method was developed for the determination of risedronate, a bisphosphonate drug important for the treatment of a variety of bone diseases, in raw material and pharmaceutical formulations. The proposed method is based on complex formation between risedronate and Cu (II) ions in acetate buffer of pH5.5. The optimum conditions for this reaction were ascertained and a spectrophotometric method was developed for the determination of risedronate in concentration range of 2-40 μg/mL with detection limit of 0.03 μg/mL (9.51 × 10^-8^ mol/L). The molar absorbtivity was 8.00 × 10^3^ l/mol/cm. The method was successfully applied for the determination of risedronate in tablet dosage form with mean percentage recovery of 101.04 ± 0.32. The results obtained were favorably compared with those obtained by the comparison method. Furthermore, the proposed method was applied for content uniformity testing of risedronate tablets.

## INTRODUCTION

**Figure 1:**
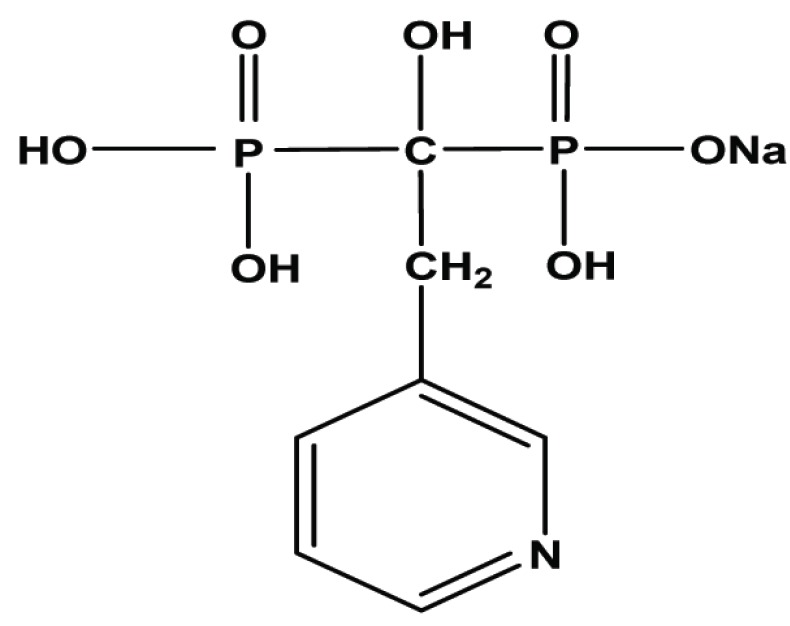
Chemical structure of risedronate sodium.

Bisphosphonates are analogues of pyrophosphate, in which the central oxygen atom is replaced by a carbon atom with two further substituents. Like pyrophosphates, they have strong affinity for bone. Risedronate sodium (sodium trihydrogen [1-hydroxy-2-(3-pyridyl) ethylidene] diphosphonate) (Fig. 1) is a pyridinyl bisphosphonate that induces remission in patients with Paget's disease (1). It is given in bone disorders in which excessive bone resorption is a problem, such as Paget's disease of bone and osteoporosis. The clinical utility of bisphosphonates resides in their ability to inhibit bone resorption. The mechanism by which this antiresorptive effect occurs is not completely known, but it is thought that the bisphosphonate becomes incorporated into bone matrix and is imbibed by osteoclasts during resorption (1, 2).

Risedronate is a non-official drug: it is not a subject of a monograph in any pharmacopoeia. Several methods have been applied in the literature for the determination of risedronate. The techniques used in this connection include spectrophotometry (3), liquid chromatography-tandem mass spectrometry (4), enzyme linked immunosorbent assay (5) and ion-pair high-performance liquid chromatography (6-9). All these methods are time consuming and require highly sophisticated instrumentation. This led us to study the reaction of risedronate with Cu (II) ions in an effort to develop a simple, rapid and sensitive method for its determination in dosage forms.

Metal complexing ability of bisphosphonates is a very important character of this group of compounds (10-14). This character was utilized to develop a method for determination of risedronate in bulk powder and pharmaceutical formulations. The proposed method based on complex formation between risedronate and copper (II) sulfate in 5 mM acetate buffer of pH5.5, the resulting complex is then quantified spectrophotometrically at λ_max_ 264 nm. This study describes an accurate, sensitive, more conventient, and less time consuming spectrophotometric method for rapid determination of risedronate in raw material and in pharmaceutical formulations. The advantages of the proposed method over already existing methods are rapidness, simplicity and inexpensiveness.

## EXPERIMENTAL

### Instruments

UV-1601, Shimadzu recording spectrophotometer (P/N 206-67001) equipped with a pair of 1 cm quartz matched cells used for all spectral measurments. Absorption spectra of samples are recorded between 200-400 nm.

A consort NV P901 pH meter was used for checking the pH of buffer solutions.

### Reagents and Materials

All the reagents used were of analytical grade and distilled water is used throughout the work.

Pure sample of risedronate sodium with a purity of 100.41% (3) (Batch # FRS/001/06-07) was kindly provided by Alkan Pharma CO., Cairo, Egypt.

Copper (II) sulfate, CuSO_4_·5H_2_O, was obtained from Merck (Darmstadt, Germany), 2.5 mM solution was prepared.

Sodium acetate trihydrate and glacial acetic acid were purchased from Merck (Darmstadt, Germany). Acetate buffer pH5.5 is prepared by adjusting the pH of 5 mM sodium acetate solution with few drops of glacial acetic acid.

Tablets containing drug (Actonel^®^ tablets Batch # 406767, labeled to contain 5 mg of risedronate sodium/tablet, product of Aventis Pharma) were obtained from commercial sources in the local market.

### Stock Solution

Standard stock solution of risedronate sodium was prepared by dissolving 50.0 mg of the drug in 100 mL distilled water. This solution was stable for at least 10 days when stored in the refrigerator and protected from light. More dilute solutions were obtained by appropriate dilution.

### General Recommended Procedures

**Procedure for calibration graph.** Transfer accurately measured aliquots of the stock solution into a series of 25-ml volumetric flasks so that the final concentration will be in the range of 2-40 μg/mL. To each flask 2.5 mL of acetate buffer and 2 mL of 2.5 mM copper (II) sulfate solution were added. The flasks were shaken well and completed to volume with distilled water. The absorbance of the formed copper (II) complex is measured at 264 nm against a reagent blank prepared in the same way without the addition of risedronate. To obtain the standard calibration graph, the values of the absorbance were plotted against the final concentration in μg/mL. Alternatively, the regression equation was derived.

**Procedure for determination of the studied drug in dosage form.** Actonel^®^ tablets are first decoated by peeling. An accurately weighed quantity of the mixed contents of 10 pulverized tablets equivalent to 25.0 mg of the drug was transferred into a 50 mL volumetric flask, and dissolved in distilled water. The contents of the flask were sonicated for 15 min and the remaining residue was removed by filteration; and the above procedure was followed. The nominal contents were calculated either from the previously plotted calibration graph or using the corresponding regression equation.

### Stoichiometric Relationship

The mole ratio method (16) employed a 2 mM solution of both the drug and the reagent under consideration (CuSO_4_·5H_2_O) to determine the stoichiometry of Cu-risedronate complex. In this method a series of solutions was prepared in which the concentration of the ligand is kept constant and that of the metal ion is varied. The absorbance of the solutions are measured at 264 nm and plotted versus the ratio of the variable.

## RESULTS AND DISCUSSIONS

### Optimization of Experimental Conditions

Factors affecting the complex formation and its stability were carefully studied and optimized.

**Figure 2:**
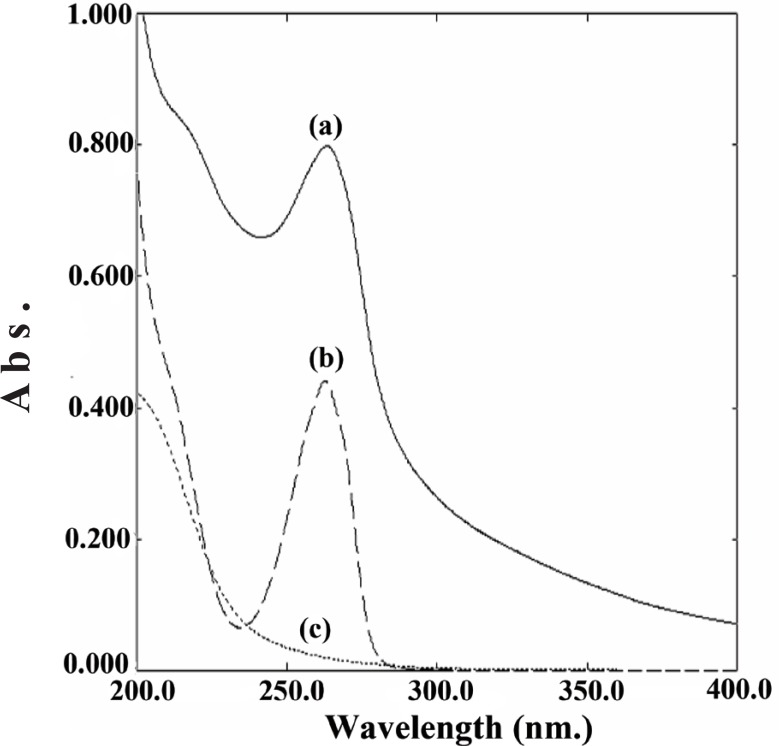
Spectra of: (a) complex of risedronate (30 μg/mL) with CuSO_4_·5H_2_O (2 mL of 2.5 mM) in acetate buffer (pH5.5); (b) risedronate (30 μg/mL) in acetate buffer (pH5.5); (c) CuSO_4_·5H_2_O (2 mL of 2.5 mM) in acetate buffer (pH5.5).

**Spectra.** The absorption spectrum of Cu-risedronate complex was recorded in the wavelength range from 200 to 400 nm (Fig. 2). The absorption spectra of risedronate and copper (II) sulfate are also given in Fig. 2. Risedronate sodium has maximum absorbance at 262 nm in acetate buffer. On addition of CuSO_4_·5H_2_O, a hyperchromic shift occurs permitting sensitive determination of risedronate.

**Investigation of complex formation in different media.** The complex formation between Cu (II) ions and risedronate was investigated in different media (acidic medium, alkaline medium and acetate buffer). Different acids such as HClO_4_, H_2_SO_4_, HNO_3_, HCl, and acetic acid were tested. It was found that no complexation occurs using any of these acids. While, in alkaline medium the metal ion precipitated. On the other hand, Cu-risedronate complex formed when acetate buffer is used. The absorption spectra for Cu-risedronate complexes using different molarities (0.005-0.1 M) of acetate buffer reveal that there’s no relation between buffer concentration and complex formation. 5 mM acetate buffer was used in all further experiments.

**Figure 3:**
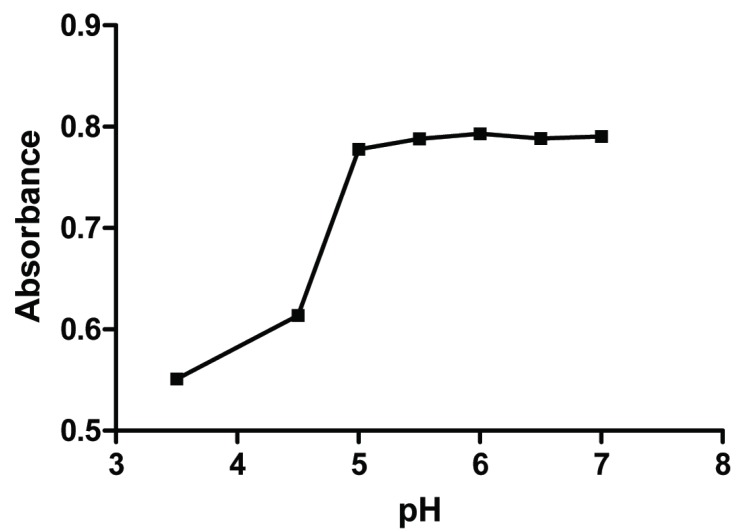
Effect of pH of 5 mM acetate buffer on the absorbance of the formed Complex of risedronate (30 μg/mL) with Cu (II) ions.

**Effect of pH.** The effect of pH on complex formation was investigated in the range 3.5-7 using 5 mM acetate buffer. It is noted that, by increasing the pH the absorbance increased up to pH5, where the absorbance reached the maximum value and no further increase was obtained (Fig. 3). The absorbance of the formed complex increased up to 2 mL of the buffer solution, after that no further increase was obtained. 2.5 mL of 5 mM acetate buffer solution of pH5.5 was chosen as optimal for all further experiments.

**Figure 4:**
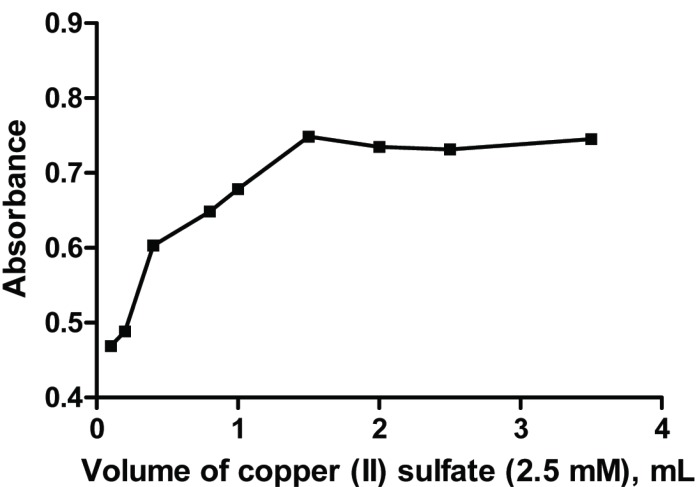
Effect of volume of 2.5 mM Copper (II) sulfate solution on the absorbance of the formed complex of risedronate (30 μg/mL) with Cu (II) ions.

**Effect of Copper (II) sulfate concentration.** Maximum absorbance was achieved with volume of 1.5 mL of 2.5 mM CuSO_4_·5H_2_O, larger volumes of the metal ion has no effect on the absorbance (Fig. 4). 2 mL of the metal ion solution was chosen to ensure maximum absorbance.

**Effect of diluting solvent.** The effect of diluting solvent on the formation and stability of Cu-risedronate complex was investigated. Different diluting solvents such as water, methanol, acetone, acetonitrile, dimethylformamide and dimethylsulfoxide were tested. It was found that maximum absorbance intensity obtained using water as diluting solvent. Methanol caused a slight decrease in the absorbance intensity, while the other solvents caused dramatic sharp decreases in absorbance intensity. Water was therefore selected as the best diluting solvent. Using water adds to the advantages of the proposed method with regard to cost and simplicity.

**Effect of temperature.** Different temperatures varying from ambient to boiling temperature were studied. It was found that heating did not enhance the complexation reaction between risedronate and Cu (II) ions. All further experiments carried out at room temperature.

**Effect of surfactants and sensitizers.** Different sensitizers (quinine, eosin and rhodamine-B), at concentrations of 5 μg/ml were tested by adding to the reactants mixture before measuring the absorbance intensity at 264 nm. It was found that none of these sensitizers had a significant effect on the absorbance intensity of the formed complex as shown in Table 1. In the same manner, the effect of surfactants on the formation of Cu-risedronate complex was studied. Different surfactants (cetrimide, gelatin and sodium lauryl sulfate) at three concentrations, 2.5, 7.5 and 15 μg/ml, were tested by adding to the reaction mixture prior measuring the absorbance intensity of the formed complex at its λ_max_. All tested surfactants had a negligible effect on complex formation (Table 1).

**Table 1:** Effect of surfactants and sensitizers on the performance of the proposed method

Surfactant/Sensitizer	Concentration of surfactant/sensitizer (μg/mL)	Absorbance

No sensitizer		0.7690
Quinine-HCl	5	0.8181
Rhodamine-B	5	0.7461
Eosin	5	0.7759
No surfactant		0.7884
Sodium lauryl sulfate	2.5	0.7383
	7.5	0.7693
	15	0.7256
Cetrimide	2.5	0.7441
	7.5	0.8009
	15	0.7377
Gelatin	2.5	0.7748
	7.5	0.8234
	15	0.8318

**Effect of time on the formation and stability of the formed complex.** The effect of time on the absorbance of drug-metal complex was investigated. It was found that the complex formation is instantaneous and the formed complex is stable for at least 90 min.

### Molar Ratio

**Figure 5:**
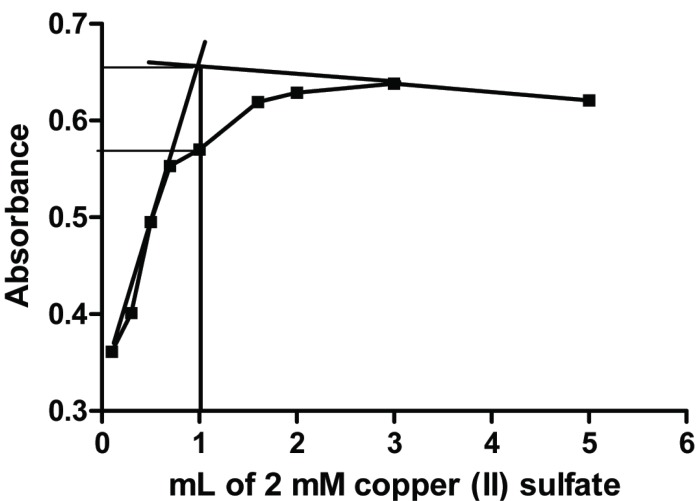
Mole ratio of risedronate and Cu (II) ions (2 mM for each).

The plot obtained by the molar ratio method (16) indicated that Cu (II) ions and risedronate form complex in a molar ratio of 1:1 (Fig. 5).

### Method Validation

**Figure 6:**
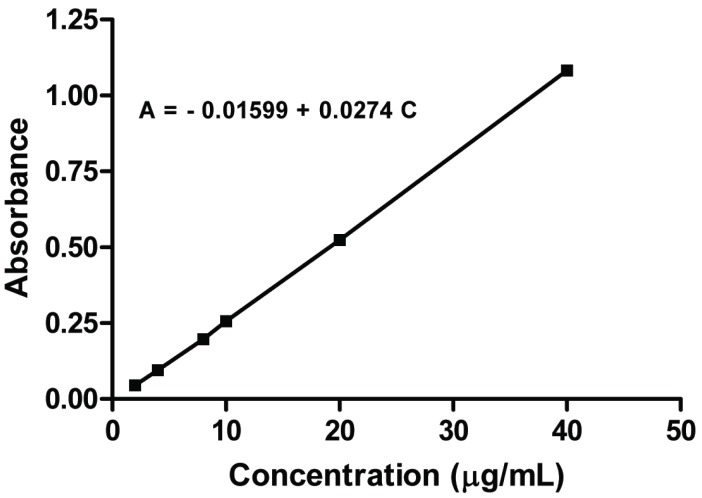
Standard calibration curve for determination of risedronate sodium by the proposed method.

**Concentration range and calibration graph**. The calibration graph (Fig. 6) obtained by plotting the values of the absorbance versus the final concentration were found to be rectilinear over the concentration range cited in Table 2. Linear regression analysis of the data gave the following equation:

A=−0.01599+0.0274 C  r=0.9999

where A is the absorbance at 264 nm, C is the concentration in μg/mL and r is the correlation coefficient.

**Table 2:** Performance data for the proposed method

Parameter	Data

Concentration range (μg/mL)	2-40
Limit of detection (LOD) (μg/ml)	0.03
Limit of quantification (LOQ) (μg/mL)	0.09
Correlation coefficient (r)	0.9999
Slope	0.0274
Intercept	-0.01599
S_y/x_	6.12 × 10^-4^
S_a_	2.41 × 10^-4^
S_b_	1.93 ×10^-5^
RSD,%	1.67
% Error	0.75
ε(l/mol./cm.)	8.00 × 10^3^

S_y/x_, Standard deviation of the residuals; S_a_, Standard deviation of the intercept; S_b_, Standard deviation of the slope; % Error, % RSD/√n; ε, Molar absorbtivity.

Statistical analysis of the data gave small values for the standard deviations of the residuals (S_y/x_), the standard deviation of the intercept (S_a_), the standard deviation of the slope (S_b_), and the % relative error (% Er) as shown in Table 2.

**Limit of quantitation and limit of detection.** The limit of quantitation (LOQ) was determined by establishing the lowest concentration that can be measured according to ICH Q2 (R1) recommendation (17) below which the calibration graph is non-linear and was found to be 0.09 μg/mL. The limit of detection (LOD) was determined by evaluating the lowest concentration of the analyte that can be readily detected and was found to be 0.03 μg/mL (9.51 × 10^-8^ M/L).

LOQ and LOD were calculated according to the following equations (17):

LOQ=10 S_a_/b

LOD=3.3 S_a_/b

where S_a_ is the standard deviation of the intercept of regression line, and b is the slope of the regression line.

**Accuracy and precision.** The intra-day precision and accuracy of the assay were measured by analyzing three concentrations in one day. Also, the inter-day precision and accuracy were determined over three successive days by analyzing the same concentrations. The results obtained for both the intra and inter-day precision and accuracy are abridged in Table 3.

**Table 3:** Accuracy and precision data for risedronate using the proposed method

Parameter	Intra-day precision			Inter-day precision		
	Concentration taken (μg/ml)	Concentration found (μg/ml)	Recovery %	Concentration taken (μg/ml)	Concentration found (μg/ml)	Recovery %

	4.00	3.984	99.60	4.00	4.062	101.55
	10.00	9.814	98.14	10.00	9.896	98.96
	20.00	19.861	99.31	20.00	19.889	99.45
x ± SD			99.02 ± 0.77			99.99 ± 1.38
%RSD			0.78			1.38
%Er			0.45			0.80

Each result is the average of three separate determinations.

**Robustness of the method.** The robustness of the procedure adopted in the proposed method is demonstrated by the constancy of the absorption intensity with minor changes in the experimental parameters such as the volume of 5 mM CuSO_4_·5H_2_O, 2 ± 0.5 mL, the change in the pH of acetate buffer, 5.5 ± 0.5, and the volume of acetate buffer, 2.5 ± 0.5 mL. These minor changes that may take place during the experimental operation did not affect the absorption intensity indicating the excellent robustness of our proposed method.

### Applications

**Application of the Proposed Method to the Analysis of the Studied Drug in its Commercial Tablets.** The proposed method was applied to the determination of risedronate in its tablets. The % recovery of the studied drug compared with that obtained by the comparison method (3) was given in Table 4 and Table 5. The comparison method involved the spectrophotometric determination of the studied drug by oxidation with ceric sulfate in 0.5 M sulfuric acid and subsequent measurement of the excess unreacted cerium (IV) sulfate at 320 nm.

**Table 4:** Application of the proposed and comparison methods to the determination of risedronate in pure form

Parameter	The proposed method			Comparison method (3)	
	Concentration taken (μg/ml)	Concentration found (μg/ml)	Recovery %	Concentration taken (μg/ml)	Recovery %

Data	4.00	4.073	101.83	4.00	99.90
	8.00	7.795	97.44	8.00	101.38
	10.00	9.952	99.52	10.00	99.96
	20.00	19.726	98.63		
	40.00	40.109	100.27		
x ± S.D.			99.54 ± 1.66	100.41 ± 0.84	
t			0.828 (2.447)^a^		
F			3.905 (19.25)		

Each result is the average of three separate determinations.

a Values between brackets are the tabulated t and F values, at *p*=0.05 (15).

**Table 5:** Application of the proposed and comparison methods to the determination of risedronate in dosage form

Parameter	The proposed method		Comparison method (3)	
	Concentration taken (μg/ml)	Recovery %	Concentration taken (μg/ml)	Recovery %

Actonel^®^ tablets 5 mg/tablet))	10.00	100.87	4.00	100.38
	20.00	101.41	8.00	99.95
	30.00	100.84	10.00	102.27
x ± S.D.	101.04 ± 0.32		100.87 ± 1.23	
t	0.231 (2.776)^a^			
F	14.774 (19.00)			

Each result is the average of three separate determinations.

a Values between brackets are the tabulated t and F values, at *p*=0.05 (15).

Statistical analysis (15) of the results obtained by the proposed and comparison method (3) using Student’s *t*-test and variance ratio revealed no significant difference between the performance of the two methods regarding the accuracy and precision.

**Application of the Proposed Method to Content Uniformity Testing of Actonel^®^ tablets.** The proposed method proved to be suitable for the content uniformity test, where a great number of assays on individual tablets are required. Data presented in Table 6 indicate that the proposed method can accurately and precisely quantitate risedronate in its commercially available tablets. The mean percentage (with R.S.D.) of the labelled claim found in ten tablets was 99.64 (2.13%) which fall within the content uniformity limits specified by the USP XXX (18).

**Table 6:** Results of content uniformity testing of Actonel^®^ tablets using the proposed method

Parameter	Percentage of the label claim

Tablet No. 1	100.84
Tablet No.2	100.28
Tablet No.3	96.29
Tablet No.4	99.95
Tablet No.5	98.08
Tablet No.6	99.83
Tablet No.7	101.39
Tablet No.8	96.20
Tablet No.9	102.53
Tablet No.10	100.96
Mean (x)	99.64
± S.D.	2.12
% RSD	2.13
% Error	0.67
Acceptance value (AV) (18)	5.09
Max. Allowed AV (LI) (18)	15.00

## CONCLUSION

The spectrophotometric method described in this article was found to be rapid, simple and sensitive and therefore could be applied for the determination of risedronate in the bulk drug and in risedronate tablets. The results confirm the suitability of the proposed method for the precise analysis of risedronate. Since this method is rapid, simple and no expensive laboratory technique is needed, it can be used for routine analyses and quality control of risedronate in pharmaceutical dosage forms. Furthermore, the proposed method was extended to content uniformity testing of risedronate in tablet dosage form.

However, as a non-separative method, its main disadvantage is the inability to differentiate among degradation products and risedronate related compounds. Hence, the proposed method cannot be useful in stability testing and determination of impurities.
